# Early diagnosis of brain tumours using a novel spectroscopic liquid biopsy

**DOI:** 10.1093/braincomms/fcab056

**Published:** 2021-03-30

**Authors:** Paul M Brennan, Holly J Butler, Loren Christie, Mark G Hegarty, Michael D Jenkinson, Catriona Keerie, John Norrie, Rachel O’Brien, David S Palmer, Benjamin R Smith, Matthew J Baker

**Affiliations:** Translational Neurosurgery, Centre for Clinical Brain Sciences, University of Edinburgh, Edinburgh EH4 2XU, UK; ClinSpec Diagnostics Limited, Royal College Building, Glasgow G1 1XW, UK; ClinSpec Diagnostics Limited, Royal College Building, Glasgow G1 1XW, UK; ClinSpec Diagnostics Limited, Royal College Building, Glasgow G1 1XW, UK; Institute of Translational Medicine, University of Liverpool & The Walton Centre NHS Foundation Trust, Liverpool L9 7LJ, UK; Edinburgh Clinical Trials Unit, Usher Institute—University of Edinburgh, Edinburgh EH16 4UX, UK; Edinburgh Clinical Trials Unit, Usher Institute—University of Edinburgh, Edinburgh EH16 4UX, UK; Emergency Medicine Research Group (EMERGE), Royal Infirmiry of Edinburgh, Edinburgh EH16 4SA, UK; ClinSpec Diagnostics Limited, Royal College Building, Glasgow G1 1XW, UK; Department of Pure and Applied Chemistry, Thomas Graham Building, University of Strathclyde, Glasgow G11XL, UK; ClinSpec Diagnostics Limited, Royal College Building, Glasgow G1 1XW, UK; ClinSpec Diagnostics Limited, Royal College Building, Glasgow G1 1XW, UK

**Keywords:** brain, cancer, diagnosis, liquid biopsy, spectroscopy

## Abstract

Early diagnosis of brain tumours is challenging and a major unmet need. Patients with brain tumours most often present with non-specific symptoms more commonly associated with less serious diagnoses, making it difficult to determine which patients to prioritize for brain imaging. Delays in diagnosis affect timely access to treatment, with potential impacts on quality of life and survival. A test to help identify which patients with non-specific symptoms are most likely to have a brain tumour at an earlier stage would dramatically impact on patients by prioritizing demand on diagnostic imaging facilities. This clinical feasibility study of brain tumour early diagnosis was aimed at determining the accuracy of our novel spectroscopic liquid biopsy test for the triage of patients with non-specific symptoms that might be indicative of a brain tumour, for brain imaging. Patients with a suspected brain tumour based on assessment of their symptoms in primary care can be referred for open access CT scanning. Blood samples were prospectively obtained from 385 of such patients, or patients with a new brain tumour diagnosis. Samples were analysed using our spectroscopic liquid biopsy test to predict presence of disease, blinded to the brain imaging findings. The results were compared to the patient’s index brain imaging delivered as per standard care. Our test predicted the presence of glioblastoma, the most common and aggressive brain tumour, with 91% sensitivity, and all brain tumours with 81% sensitivity, and 80% specificity. Negative predictive value was 95% and positive predictive value 45%. The reported levels of diagnostic accuracy presented here have the potential to improve current symptom-based referral guidelines, and streamline assessment and diagnosis of symptomatic patients with a suspected brain tumour.

## Teaser

Brennan et al. show a rapid, low-cost blood test can identify which patients with suspected brain tumour to prioritize for diagnostic imaging, reducing time to diagnosis. The test is more than 90% sensitive for the most aggressive tumours. Earlier diagnosis can provide health and economic benefits.

## Graphical Abstract

**Graphical Abstract:**
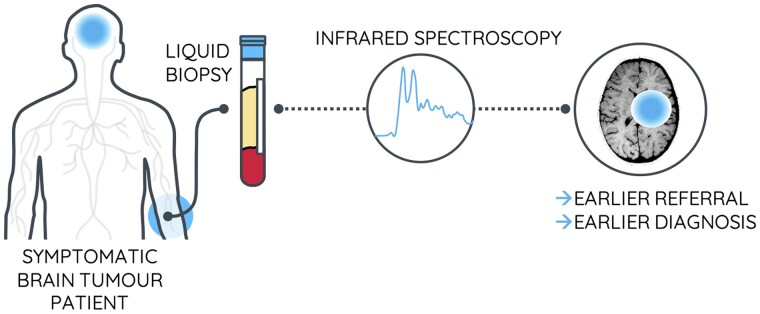

## Introduction

Identifying which patients with symptoms of a possible brain tumour should have urgent brain imaging is challenging. In the UK, only 1% of adult brain tumours are diagnosed through the emergency ‘Two Week Wait’ referral pathway, where general practitioners refer patients for urgent secondary care assessment because of a suspected tumour.^1^^,^^2^ For 19% of patients with a brain tumour, diagnostic imaging was accessed following routine primary care referrals, and almost two-thirds of patients are only diagnosed when they present to the emergency department.^3^^,^^4^ Many of these emergency patients will have previously seen a general practitioner and 38% of patients with a brain tumour saw a general practitioner five times before diagnosis. This suggests that there are potential missed opportunities for earlier diagnosis.^5^

Symptom-based referral guidelines perform poorly at identifying which patients in primary care should be referred for brain imaging for suspected tumour.^6^ Whilst presentation with more severe neurological symptoms usually results in rapid access to diagnostic imaging, most people with a brain tumour present with non-specific symptoms, such as headache, which are more likely associated with a non-tumour diagnosis and are diagnostically challenging. Brain tumours are rare, so a non-tumour diagnosis is much more likely in a primary care setting,^7^ which often leads to a diagnostic delay of several weeks for people with a brain tumour.^8^ In the symptomatic referred population, there is a reported prevalence of brain tumours between 1% and 3%.^9–11^

A study of symptom-based referral pathways for suspected brain tumour reported a positive predictive value (PPV) of 2.8% for severe red flag symptoms in terms of detecting a brain tumour on subsequent brain imaging.^9^ Headache alone as an indicator of brain tumour has a reported PPV as low as 0.1%, and only increases (7.2%) when considered with more severe cognitive symptoms over longer periods of time.^6^ There is a clear need for new tests to support brain tumour diagnosis and to reduce diagnostic delay.

We previously reported a rapid, low-cost, spectroscopic liquid biopsy approach that has the potential to improve identification of which patients in a population with non-specific symptoms are likely to have a brain tumour.^12^ The spectroscopy test analyses patient blood serum and is based upon the interaction of infrared (IR) light with all molecules present within the patient blood serum, generating a ‘biological signal’ of the sample.^13^^,^^14^ This signal is then classified using a diagnostic algorithm to predict cancer or non-cancer. Rather than being limited by analysis of single biomarkers, this signal is representative of the full biochemical profile of the serum droplet. This machine learning algorithm has been trained on a database of known disease and control patients to learn the signals of disease.^15^^,^^16^ Integrating this simple, rapid, low-cost blood test into the existing diagnostic pathway would lower the threshold for suspecting a brain tumour and permit earlier and more effective triage of patients for medical imaging, expediting assessment for patients most likely to have a brain tumour and helping to ‘rule out’ a brain tumour diagnosis in others, reducing anxiety and the need for urgent imaging. Early health economic assessment of the technology demonstrated that this blood test would fall within the cost-effectiveness threshold.^17^

We conducted the brain-early detection single-centre cohort study to assess the diagnostic accuracy of our spectroscopic liquid biopsy on symptomatic patients referred for brain imaging from primary care with suspected brain tumour, and on patients with a new brain tumour diagnosis. We have previously reported values of sensitivity and specificity for this test based upon retrospective studies using biobank sample of cancer versus healthy controls.^15^^,^^18^ In this brain-early detection cohort, we expect to see an attenuation of overall diagnostic performance as the test is applied to a symptomatic patient population. An interim analysis of these 104 patients from this study was reported previously.^12^

## Materials and methods

### Patient recruitment

Ethical approval for the brain-early detection study was granted by Lothian Research and Ethics Committee (15/ES/0094). Two related cohorts of patients were eligible for inclusion, encompassing different access points on the cancer diagnostic pathway:

- Cohort 1—Symptomatic, referred population Patients aged 16 or over referred within the pre-existing direct access computed tomography pathway available to primary care doctors in National Health Service Lothian (UK) to exclude significant intracranial pathology. Urgent (same-day) brain imaging referrals are not included in this pathway. All direct access computed tomography brain imaging is performed in a single neuroradiological department at the Department of Clinical Neurosciences, Western General Hospital, Edinburgh, Scotland.
- Cohort 2—New brain tumour diagnosis population Patients aged 16 years or older with a recent diagnosis of a primary or recurrent brain tumour referred to the Department of Clinical Neurosciences for management were also eligible.

For both cohorts, patients were recruited in secondary care facilities; (i) prior to brain scan for Cohort 1, (ii) prior to surgical intervention for Cohort 2. All patients gave written informed consent. Blood samples were obtained during routine venepuncture using Sarstedt (Germany) Monovette 7.5 ml Serum Gel Z collection tubes and were anonymized for processing and analysis. Each sample was inverted eight times, and allowed to clot for a minimum of 1 h. Samples were centrifuged at 2200 g for 15 min (or equivalent) and stored at −80°C until analysis. Patient and sample details, including symptoms and concomitant drug treatments (based on a short list of common medications), were entered into an online clinical database designed by the Edinburgh Clinical Trials Unit to meet sufficient standards of data integrity and safety.

### Study design

We assessed the acceptable clinical performance of the liquid biopsy test based upon previously reported levels of sensitivity (93.75%) from a retrospective study of biobank samples^18^; there are no existing biomarker tests for brain tumours in clinical use against which we could compare the performance of our test. Based on the reported sensitivities and specificities, and approximate prevalence level in the referred population, we determined that our study cohort should total approximately 600 patients to achieve a conservative sensitivity of 90%, estimated to a clinically acceptable precision of ±17% (half-width confidence interval). Prevalence in the population has been reported between 1% and 3%, and the upper reported limit was used as a comparator in this study.^19^ As part of the study, a specified interim analysis was included to allow the opportunity to test assumptions behind the sample size calculations. If the precision of a smaller cohort was such that it provided sufficient symptomatic data for the next phase of algorithm development, recruitment could be ceased. For the interim analysis, we planned to assess the cohort precision at approximately 400 patients. The authors conducted a preliminary interrogation of the data after recruitment and analysis of 104 patients, whilst remaining blind to the clinical information; details of this analysis were included in the article by Butler et al.^12^

### Spectroscopic liquid biopsy

**Figure 1:**
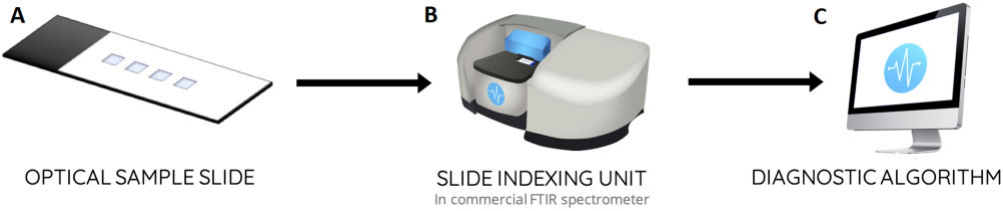
The spectroscopic liquid biopsy platform. (**A**) Optical sample slides containing four wells, for triplicate sample measurements and a single background measurement; (**B**) slide indexing unit to integrate slides with a commercially available FTIR spectrometer; and (**C**) diagnostic algorithm contained within bespoke operating software that auto-generates a disease prediction.

Patient serum samples were analysed using our spectroscopic liquid biopsy test; a platform technology based upon attenuated total reflectance-Fourier transform IR (FTIR) spectroscopy (Fig. 1). This platform adapts traditional attenuated total reflectance-FTIR spectroscopy instrumentation, into a high-throughput alternative that is suited to clinical applications and workflows. The optical sample slides replace conventional internal reflection elements which are a key component of the attenuated total reflectance-FTIR spectroscopic method, and provide a low-cost alternative that allows batch processing and disposable sampling. The slides used in this study contain four wells; three sample wells and one background well (Fig. 1A). The slide is then interfaced with commercially available FTIR spectrometers using the slide indexing unit that translates each of the sample wells across the IR beam, allowing spectral collection (Fig. 1B). A total of three spectra, per three sample wells, resulting in nine spectra per patient, are then fed into the diagnostic algorithm which generates the disease prediction (Fig. 1C). For further details, we direct the reader the publication by Butler et al.^12^

In this study, patient serum samples collected prior to brain imaging (Cohort 1) or brain tumour surgery (Cohort 2) were stored onsite at the Western General Hospital, Edinburgh, National Health Service Lothian, until date of analysis. The time-to-analysis ranged between 1 and 14 days in −80°C freezer storage, which previous investigations have demonstrated to have no impact on sample integrity, or test accuracy.^20^ Samples were allowed to thaw for up to 30 min at room temperature (20–22°C), and inverted three times to ensure sufficient mixture and thawing.

Each patient sample was prepared for spectroscopic liquid biopsy by pipetting 3 μl of serum onto three wells of the optical sample slides (Fig. 1A). Slides were then dried for 1 h, in an incubator cabinet at 35°C, permitting controlled drying. We have previously shown that controlled temperature drying allows for stable and reproducible spectral data, due to the controlled formation of drying patterns.^21^ When dried, each patient slide was then analysed using the attenuated total reflectance-FTIR spectroscopy platform, by placing the slide into the slide carriage on the slide indexing unit (Fig. 1B). In this study, a Perkin Elmer Spectrum 2 FTIR spectrometer (Perkin Elmer, US) was used to generate spectral data using locked down spectral parameters.

### Algorithm training

Following spectral acquisition, the nine spectra of each patient were analysed using our diagnostic algorithm—a machine learning model trained and tuned to accurately detect the signal of brain cancer (Fig. 1C). Machine learning approaches are essential for extracting diagnostic information from the spectral data; by eye, the average spectra of cancer and non-cancer patients are almost identical.^12^ We previously reported that this model was trained on a retrospective database of 724 known brain cancer and control patients, details of which can be found in the Supplementary Tables 1 and 2.^12^ During its training phase, this model learns the spectral indicators that are linked to brain cancer, so we can then test newly acquired spectra against the model, blinded to the disease phenotype. The diagnostic algorithm was developed over a planned iteration period to address key aspects of the model, including; spectral pre-processing, classifier choice, and classifier parameters. The final algorithm reported here is trained on 724 retrospective patients over multiple iterations and cross-validation steps, and subsequently tested on the full 385 cohort described as part of this study. All results are reported on a per patient basis.

### Disease prediction

A prediction is generated for each of the nine spectra acquired from each patient, and a consensus prediction determined which gives the overall result. The consensus was taken as a maximum vote such that a final prediction of ‘cancer’ was made if five or more spectra agreed in their prediction of ‘cancer’. Receiver operating characteristic curves were generated by altering the probability threshold of the algorithm between cancer and non-cancer and noting the relative sensitivity and specificity.

In this study, disease predictions were generated for the anonymized cohort of patients and were submitted for review as part of the interim analysis. Throughout this process, true patient disease class was left blind to all analysts and operators, and lead clinician Dr Paul Brennan was responsible for unblinding and reporting test performance against CT as a reference standard. The reference standard in this study was CT imaging, to confirm or refute evidence of central nervous system tumours, followed by diagnosis by biopsy if clinically indicated. In a previous review of 3000 patient brain CT scans undertaken through the same open access referral pathway for suspected brain tumours as detailed in this study, we reported that no brain tumour diagnoses were missed on CT alone with up to 5 years of follow-up for each patient.^22^ Hence CT was an adequate gold standard investigation in this study.

### Data availability

Clinical data, summary test statistics and study details are available upon request.

## Results

### Recruitment overview

**Figure 2:**
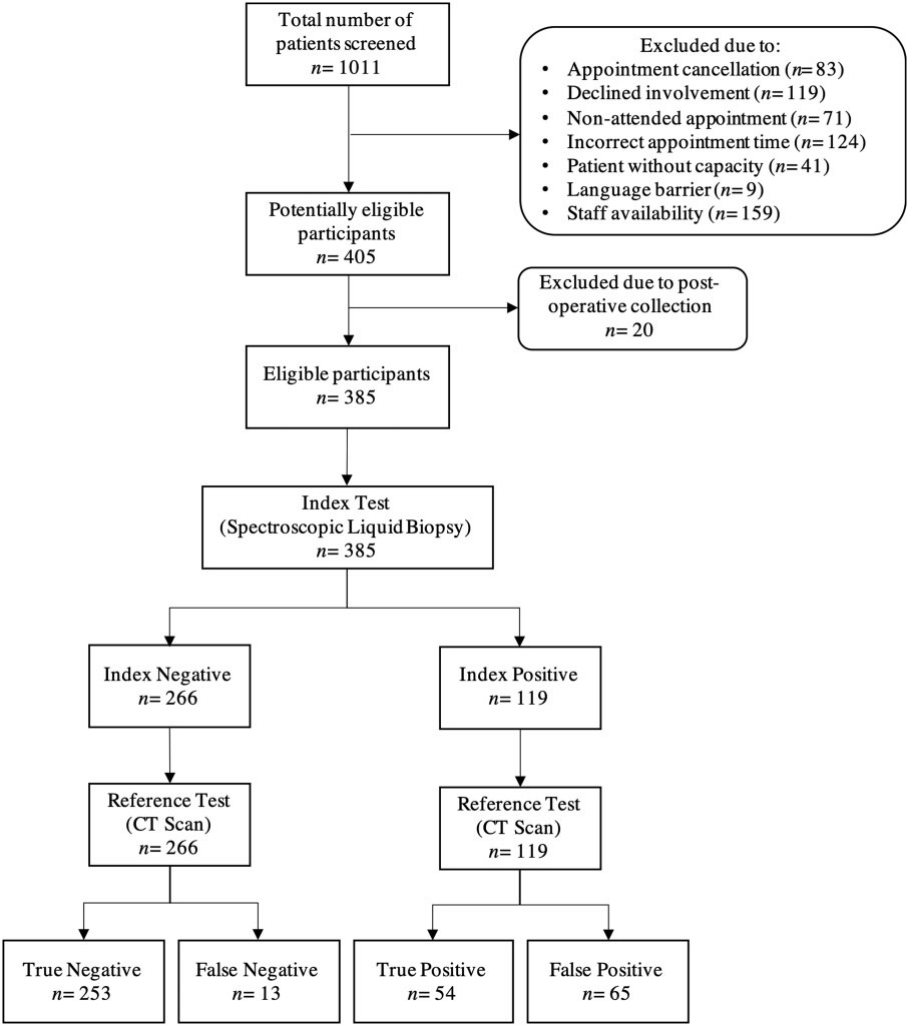
**Flow of participants through brain-early detection study according to standards for reporting diagnostic accuracy**.

A total of 405 patients were assessed for inclusion in this study, with a final eligible cohort of 385 patients reported (Fig. 2). From January 2018 to February 2019, a total of 1011 patients were screened for eligibility based primary care referral pathway for brain imaging. Reasons for not participating in the study included appointment cancellation (*n* = 83), patient declined study involvement (*n *= 119), patient did not attended appointment (*n* = 71), patient attended appointments at incorrect time (*n* = 124), patient judged to not have capacity (*n* = 41), language barrier to consent (*n* = 9) and also staff availability (*n* = 159).

Serum samples from the first 405 consecutively recruited patients were assessed. Twenty patient samples were excluded where samples had been taken during, rather than before, surgical intervention. The age and gender breakdown of the cohort are presented in the Supplementary Table 1. In the eligible cohort, 385 patients samples were analysed in the pre-specificed interim anlaysis, of which 66 patients had a confirmed brain tumour, resulting in an overall prevalence of 17% in this cohort (Table 1). Of the patient samples analysed, three brain tumours were identified through the Cohort 1—direct access computed tomography pathway, and all other brain tumours were obtained from Cohort 2. The largest proportion of the patients with a brain tumour were diagnosed with glioblastoma, the most common and most malignant brain tumour. Of the metastatic tumours to the brain, the most common originated in the lung (*n* = 9), with the remainder orginating from breast (*n* = 1), ovarian (*n* = 1), endometrial (*n* = 1), rectal (*n* = 1), testicular (*n* = 1) and carcinosarcoma (*n* = 1) tissues, with a single tumour of unknown primary (*n* = 1).

**Table 1:** Patient breakdown per disease classification in eligible population

No tumour	318
Glioblastoma	34
Metastatic	16
Meningioma	5
Anaplastic astrocytoma	3
Anaplastic oligodendroglioma	2
Pituitary adenoma	1
Schwannoma	1
Subependymoma	1
Medulloblastoma	1
Diffuse astrocytoma	1
Ependymoma	1
Oligodendroglioma	1
Total	385

### Diagnostic performance

Test performance was determined by comparison of the disease prediction generated by the spectroscopic liquid biopsy test, against the reported diagnosis from CT brain imaging (Table 2). The performance of our test in identification of which blood samples came from patients with any brain tumour was 81% sensitivity and 80% specificity (Table 3). Test performance was also assessed for identification of the most frequent tumour, glioblastoma. Thirty-three tumours were confirmed histologically as glioblatoma. Our test reported 30 true positives and 3 false negatives, giving a sensitivity of 91%.

**Table 2:** Confusion matrix of the spectroscopic liquid biopsy predictions versus confirmed diagnosis via CT scan

		Imaging		
		Positive	Negative	Total
Test	Positive	54	65	119
	Negative	13	253	266
	Total	67	318	385

**Table 3:** Diagnostic performance of the spectroscopic liquid biopsy

		95% CI	
	Reported	Lower	Upper
Sensitivity	81%	71%	90%
Specificity	80%	75%	84%
Prevalence	17%	14%	21%
PPV	45%	36%	54%
NPV	95%	93%	98%
Accuracy	80%	76%	84%

NPV, negative predictive value; PPV, positive predictive value.

The complete diagnostic performance of the interim analysis study cohort is summarized in Table 3. Based on the sensitivity (81%) and prevalence (17%) associated with this cohort, the sample size of 385 patients was determined to achieve a level of precision (measured as the half-width of the confidence interval) of ±9.5%. If this level of sensitivity and prevalence were maintained in the remaining 200 patients as determined in the study size calculation, the total sample size of 600 would achieve a level of precision of ±7.6%. These estimates of precision exceed the estimate of ±17% which formed the basis of the original sample size calculation. As there was a high level of precision from the 385 patients in the interim anlaysis cohort, this was therefore an appropriate point to report on the study, and also a significant dataset to contribute towards algorithm development.

As PPV is dependent upon prevalence of disease in the patient cohort, our enriched population yields an increased prevalence compared to a primary care setting, and thus a high PPV of 45%. When considering PPV in a primary care setting, we know from our previous analysis of open access referrals that the prevalence of brain tumours in patients referred by general practitioners for brain imaging to exclude a brain tumour is 1–3% (Zienius 2019). In that primary care context, using the upper limit of 3%, the PPV would reduce to 7.7%.

**Figure 3:**
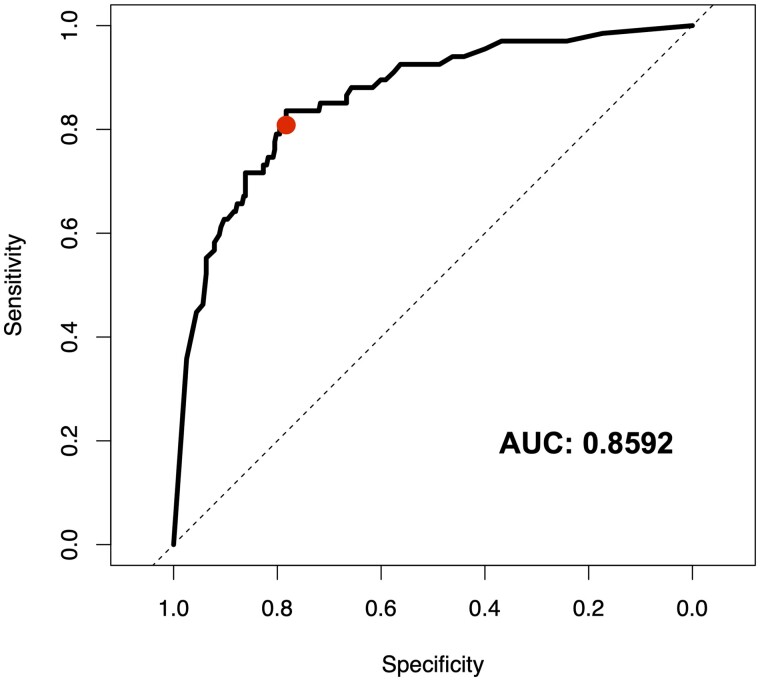
**Receiver operating characteristic curve for the spectroscopic liquid biopsy based on the symptomatic patient cohort.** The reported test performance is highlighted in red. AUC, area under the curve.

When the threshold between cancer and non-cancer is altered within the diagnostic algorithm, it is possible to observe the relationship between sensitivity and specificity of the test, and determine overall performance (Fig. 3). The area under the receiver operating characteristic curve suggests good binary classification between cancer and non-cancer, with a similar relationship between sensitivity and specificity shown. There is a slight skew towards high specificity, which is likely due to the bias in the patient cohort towards non-cancer. Interestingly, in this study, we report accuracy based upon the most balanced model between sensitivity and specificity at 81% and 80% respectively, yet a threshold change to reduce specificity a small amount could yield a most sensitive model. For example, at a specificity of 78%, it is possible to achieve a sensitivity of 84%.

### Metadata investigations

Table 4 describes the patient metadata breakdown of the 385 patients in the interim-analysis cohort. We observed the expected change in brain tumour incidence with age, and this was mirrored in variation by age of referrals with suspected cancer who transpired to not have tumours (Supplementary Table 3).

**Table 4:** Overview of available metadata from interim analysis patient cohort

Metadata		Cancer	Non-cancer
Headache	Yes	34	102
	No	32	209
	NA	3	5
Headache duration	<1 month	10	49
	<6 months	14	31
	+6 months	8	115
	NA	37	121
Headache worse in morning	Yes	9	61
	No	18	146
	NA	42	109
Headache with nausea	Yes	9	76
	No	17	133
	NA	43	107
Memory change	Yes	13	124
	No	47	185
	NA	9	7
Personality change	Yes	11	80
	No	50	230
	NA	8	6
Verbal fluency	15+	26	47
	<15	16	91
	NA	27	178
Steroids	Yes	35	50
	No	4	105
	NA	30	161
Anti-epileptic drugs	Yes	20	17
	No	49	299
Statins	Yes	17	60
	No	52	256
Anti-hypertensives	Yes	18	93
	No	51	223
Anti-thrombotics	Yes	8	45
	No	61	271
NSAIDs	Yes	6	53
	No	63	263
BMI	S. underweight	2	5
	Underweight	3	32
	Normal	37	145
	Overweight	18	91
	Obese	6	19
	S. obese	0	2
	NA	3	22

Where data were unavailable or not recorded, ‘NA’ (not available) was noted.

BMI, body mass index; NSAID, non-steroidal anti-inflammatory drugs.

Patient metadata was also used to explore the diagnoses predicted by the spectroscopic liquid biopsy test in order to understand whether there were any clinical factors which explained test performance; however, no clear patterns could be ascertained. The patient distribution of age for incorrect diagnoses mirrored correct diagnoses (Supplementary Fig. 1). Drug administration in the patient cohort was as expected; a higher proportion of patients in Cohort 2 were taking steroids, and anti-epilepsy drugs, for control of brain oedema and seizures, respectively, when compared to Cohort 1. Other drugs were evenly administered between cancer and non-cancer patients. These medications did not impact on test performance (Supplementary Table 4). Body mass index did not influence test performance.

## Discussion

We have reported the first full analysis of our study of a spectroscopic liquid biopsy test for brain tumours. We demonstrated that the test performs with 91% sensitivity for the most common brain tumour, glioblastoma, and 81% sensitivity and 80% specificity for all brian tumours. There is a clear clinical utility for this spectroscopic liquid biopsy.

A strength of this study is that patient samples were blinded before analysis. This provides an insight into how this test would perform in routine clinical practice. Most patients in our study cohort were referred from primary care for brain imaging to exclude a significant intracranial pathology, such as a brain tumour. Three patients (0.9%) referred from primary care for open access CT had a new brain tumour diagnosis, consistent with the reported 1–3% rate of brain tumours in the referred symptomatic primary care patient population.^9^^,^^19^ The overall prevalence of tumours in our study cohort was 17%, which reflects inclusion of patients with brain tumours prior to surgery. This was necessary to provide further positive events within the study to permit greater understanding of test performance and accuracy.

In this enriched patient cohort, we present a PPV of 45% for the presence of a tumour on brain imaging in patients with a positive spectroscopy test result. If we consider the true prevalence of brain tumours in the target referred symptomatic population, considered as an average prevalence of 3%, the equivalent PPV from our data equates to 7.7%. This has great promise in comparison to the performance of symptom-based referral pathways. This provides more than a 70-fold improvement in brain tumour detection compared to headache alone, and also an improvement on the performance of symptoms-based referral pathways. An examination of the NICE 2005 and Kernick referral guidelines for symptoms suggestive of brain tumour reported a PPV of 2.9 and 2.8 for the ‘symptoms related to Central Nervous System (CNS)’ and ‘red flag symptoms’, respectively.^22^ The spectroscopy test is even more beneficial in the patients with non-specific symptom groups where PPV of symptom-based referral pathways is 0.2–0.8%. The spectroscopic liquid biopsy test will effectively support clinician decision-making, allowing earlier referral for diagnostic imaging of symptomatic patients most likely to have a brain tumour. This means that positive cases can be referred earlier, but also that negative cases can also be used to rule out unnecessary referrals based on a 95% negative predictive value level.

The test performed best in patients with glioblastoma (*n* = 33), with a sensitivity of 91%. Glioblastoma is the most common and most aggressive brain tumour, so this is an important finding. Earlier diagnosis of glioblastoma will translate into better patient outcomes, for at least a proportion of patients. If the tumour is detected when it is smaller, there is greater likelihood of achieving a complete surgical resection, which is the only modifiable prognostic factor for this tumour.^23^ Surgery when the tumour is smaller will be associated with less risk of surgical morbidity. The high performance of the test for glioblastoma may reflect that glioblastoma cases were the dominant brain tumour in the 724-patient training database, constituting 53% of tumours.^12^ Fewer training samples of other tumour types may lead to inaccurate diagnosis of those less common cancers. At the next stage of algorithm development, we plan to increase numbers of non-glioblastoma brain tumours to investigate whether this improves the performance of the test for ‘all brain tumours’.

The test will be of most value to primary care doctors as a rule-out tool, supporting their clinical decision based on patient history and examination. Approximately 98% of patients presenting with symptoms in primary care suggestive of a brain tumour do not have a tumour.^9^^,^^24^^,^^25^ In this population, a test reporting a low brain tumour likelihood could support a decision to observe a patient for longer rather than refer for immediate brain imaging. If the patients symptoms then resolve, an unnecessary CT scan will have been avoided, and the patient will not have the anxiety of waiting for the scan. Brain imaging can also have a negative benefit-to-harm profile in patients with relatively minor symptoms if the imaging leads to further invasive testing because of a false positive result or incidental findings. This scenario can cause unnecessary anxiety and may cause harm from treatment for a disease which would never otherwise have presented within the patient’s lifetime, such as a small incidental meningioma.^26^

Based on the current reported levels of accuracy, a negative result is correct in 19 out of 20 patients, helping to minimize the number of unnecessary referrals to imaging facilities. In the 1 in 20 patients incorrectly reported as negative, the progression of symptoms and continued observation would ensure referral occurred soon afterwards by the assessing clinician. Use of the test as a triage tool in primary care could therefore potentially provide cost savings to the National Health Service and other health services.^17^ The direct access CT brain imaging symptom-based referral pathway utilized in this study undertakes 60 negative CT scans for every one positive scan, highlighting the number of potentially unnecessary investigations.^9^ The addition of our liquid biopsy test could reduce the number of CT scans from 60 down to as few as 12 scans for every positive diagnosis. This would ensure patients most in need of brain imaging can access it more quickly.

There are over 100 distinct classifications of brain tumour identified by World Health Organization (WHO), of which many are extremely rare, and seldom seen in a primary care setting. Our spectroscopic liquid biopsy is able to perform well across a range of the most common brain tumour types. The majority of brain tumour cases in both this patient cohort and the 724-patient retrospective dataset (used to train the diagnostic algorithm) are classed as glioblastoma, in line with expected prevalence in the population. At the next stage of algorithm development, we will add data from patients with less common brain tumours into the training dataset of the model to examine whether this enhances the overall diagnostic accuracy of the test. We will also add the data from the symptomatic population generated in this study. Future multicentre studies will reduce any demographic bias in the training set population, and should aim to reflect the target population and tumour prevalence more closely. The low incidence of brain tumours in the general population makes recruitment to a randomized study in primary care very difficult and expensive. A cohort study approach may be preferable.

## Conclusions

Earlier diagnosis of brain tumour is a major unmet clinical need. Brain tumours are rare, symptoms are often non-specific, and diagnosis requires access to expensive imaging resources. Our study has demonstrated clear clinical utility of our test in a real-world patient cohort, and provides strong evidence that the test may enable earlier diagnosis of brain tumours in clinical practice. Symptomatic patients most likely to have a brain tumour can be prioritized for brain imaging, whilst unnecessary imaging referrals can be avoided. This will improve the efficiency of the current diagnostic pathway which will provide benefits to both patients and health service providers. A liquid biopsy for these patients will mean a reduction in time-to-diagnosis and time-to-treatment, positively impacting on quality of life.

## Supplementary material

Supplementary material is available at *Brain Communications* online.

## Supplementary Material

fcab056_Supplementary_Data

## Abbreviations

FTIR =: Fourier transform infrared
IR =: infrared
PPV =: positive predictive value
