# Supplementary material for: Early diagnosis of brain tumours using a novel spectroscopic liquid biopsy
Source: Brain Commun. 2021 Mar 30;3(2):fcab056. doi: 10.1093/braincomms/fcab056 (PMC8111062; doi:10.1093/braincomms/fcab056)
Supplement: fcab056_Supplementary_Data [file fcab056_supplementary_data.docx]

**Supplementary Information**

| **Supplementary Table 1.** Patient age and gender breakdown per disease in eligible population [Male/(Female)] | | | |
| --- | --- | --- | --- |
|  |  | **Cancer M/(F)** | **Non-cancer M/(F)** |
| **Age** | **20+** | 0 (0) | 5 (14) |
|  | **30+** | 2 (3) | 16 (20 |
|  | **40+** | 4 (5) | 12 (18) |
|  | **50+** | 2 (6) | 23 (28) |
|  | **60+** | 12 (9) | 20 (44) |
|  | **70+** | 8 (5) | 35 (36) |
|  | **80+** | 4 (5) | 23 (21) |
|  | **90+** | 0 (1) | 3 (1) |

| **Supplementary Table 2.** Retrospective patient cohort breakdown with tumour classification details ^12^. | | | | |
| --- | --- | --- | --- | --- |
|  | **WHO Classification** | **Tumour Type** | **WHO Grade** | **Total** |
| ***Tumour*** |  |  |  |  |
|  | Diffuse astrocytic and oligodendroglial tumours | Glioblastoma multiforme | IV | 260 |
|  |  | Gliosarcoma | IV | 4 |
|  |  | Oligodendroglioma | II | 11 |
|  |  | Diffuse astrocytoma | II | 23 |
|  |  | Anaplastic astrocytoma | III | 10 |
|  |  | Oligoastrocytoma | II | 3 |
|  |  | Glioma | I | 7 |
|  | Other astrocytic tumours | Pilocytic astrocytoma | I | 9 |
|  |  | Pleomorphic xanthoastrocytoma | II | 1 |
|  | Tumours of the cranial and paraspinal nerves | Schwannoma | I | 14 |
|  | Ependymal tumours | Ependymoma | II | 6 |
|  | Mesenchymal, non-meningothelial tumours | Hemangiopericytoma | II/III | 2 |
|  |  | Haemanglioblastoma | I | 1 |
|  | Neuronal and mixed neuronal-glial tumours | Ganglioglioma | I | 1 |
|  | Embryonal tumours | Medulloblastoma | IV | 1 |
|  | Tumours of the pineal region | PPTID | II/III | 1 |
|  | Meningiomas | Meningioma | I | 46 |
|  | Pituitary tumours | Pituitary adenoma |  | 29 |
|  | Lymphomas | Lymphoma |  | 2 |
|  | Metastatic tumours | Metastasis |  | 56 |
| ***Control*** |  |  |  | 237 |
|  |  |  | **Total** | 724 |
| *PPTID – *Pineal parenchymal tumour of intermediate differentiation* | | | | |

| **Supplementary Table 3.** Information about the retrospective patient cohort that was used to train the diagnostic algorithm ^12^**.** | | |
| --- | --- | --- |
|  | ***Cancer*** | ***Non-Cancer*** |
| **Total** | 487 | 237 |
| **Sex (M/F)** | 280/207 | 149/84 |
| **Age Range** | 21-96 | 19-69 |
| **Average Age** | 61 | 35 |

| **Supplementary Table 4. Ratio of drug intervention between correct and incorrect diagnoses.** | | | | | | |
| --- | --- | --- | --- | --- | --- | --- |
|  | **Steroids** | **AEDs** | **Statins** | **Anti-hypertensives** | **Anti-thrombotics** | **NSAIDs** |
| **Correct Diagnosis** | 4.0 | 3.6 | 4.1 | 5.9 | 9.6 | 4.4 |
| **Incorrect Diagnosis** | 3.8 | 3.9 | 3.8 | 3.3 | 3.5 | 3.8 |

**
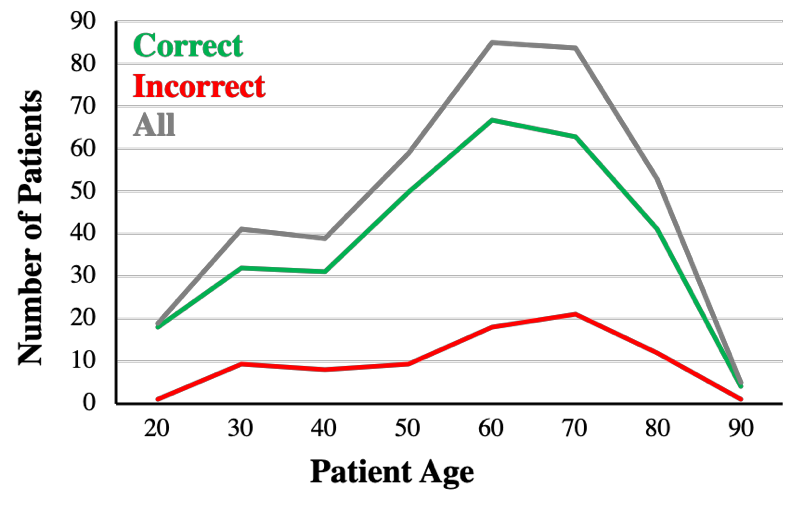
**

**Supplementary Figure 1. Patient age distribution across prediction accuracy.** Incorrect (red) and correct (green) diagnoses plotted against age frequency in patient population to determine age impact on diagnostic performance. Total age distribution is displayed in grey. Age was collected in discrete decile groups for patient anonymity.
